# Confinement-Induced Drug-Tolerance in Mycobacteria Mediated by an Efflux Mechanism

**DOI:** 10.1371/journal.pone.0136231

**Published:** 2015-08-21

**Authors:** Brilliant B. Luthuli, Georgiana E. Purdy, Frederick K. Balagaddé

**Affiliations:** 1 KwaZulu-Natal Research Institute for Tuberculosis and HIV, Nelson R. Mandela School of Medicine, University of KwaZulu-Natal, Durban, 4001, South Africa; 2 Dept. of Molecular Microbiology and Immunology, Oregon Health and Sciences University, 3181 S. W. Sam Jackson Park Rd., Mail Code L220, Portland, OR, 97239, United States of America; Centre National de la Recherche Scientifique—Université de Toulouse, FRANCE

## Abstract

Tuberculosis (TB) is the world’s deadliest curable disease, responsible for an estimated 1.5 million deaths annually. A considerable challenge in controlling this disease is the prolonged multidrug chemotherapy (6 to 9 months) required to overcome drug-tolerant mycobacteria that persist in human tissues, although the same drugs can sterilize genetically identical mycobacteria growing in axenic culture within days. An essential component of TB infection involves intracellular *Mycobacterium tuberculosis* bacteria that multiply within macrophages and are significantly more tolerant to antibiotics compared to extracellular mycobacteria. To investigate this aspect of human TB, we created a physical cell culture system that mimics confinement of replicating mycobacteria, such as in a macrophage during infection. Using this system, we uncovered an epigenetic drug-tolerance phenotype that appears when mycobacteria are cultured in space-confined bioreactors and disappears in larger volume growth contexts. Efflux mechanisms that are induced in space-confined growth environments contribute to this drug-tolerance phenotype. Therefore, macrophage-induced drug tolerance by mycobacteria may be an effect of confined growth among other macrophage-specific mechanisms.

## Introduction

Tuberculosis, caused by infection with *Mycobacterium tuberculosis* (*Mtb*) remains one of the world’s deadliest diseases, killing an estimated 1.5 million people annually [1]. Whereas drug-susceptible forms of the disease are in principle curable, the duration of treatment courses is at least 6 months and may last years [2]. Multidrug resistant TB (MDR-TB) and extensively drug resistant TB (XDR-TB) have poorer and less certain outcomes [2–4]. It is expected that shortened antituberculosis treatment regimens will improve patient adherence to treatment, and thereby foster better case management and disease control and minimize the risk of drug resistance [5, 6]. An interesting aspect of long-term chemotherapy in TB is that, whereas more than 95% of the tubercle bacilli population detectable in a patient’s sputum can be cleared in the first few days of treatment, prolonged treatment is required to eradicate the residual minority population (<5%) [7, 8] using drugs that are rapidly potent in vitro [9–11]. Overcoming this bacterial persistence is central to shortening TB treatment [12]. The transient nature of persistence—an epigenetic drug-tolerance phenotype—has pushed research in this area toward miniaturization and chip-based control using time-lapse microscopy to study the phenotypic heterogeneity within bacterial populations in situ [13–15]. Whereas these efforts have thus far investigated persistence in an extracellular context, recent studies have shown that the intracellular (or intramacrophage) mycobacterial sub-population, which makes up an essential component of human TB infection [16, 17], is significantly more tolerant to antibiotics [18, 19]. Separate studies have shown that the dimensions and diffusional characteristics of the growth environment can influence bacterial gene expression [20–22]. To investigate persistence, we characterized the growth and drug susceptibility of mycobacteria replicating in space-confined microfabricated cell culture environments (or microdialysers) to mimic the confinement experienced by mycobacteria replicating within macrophages.

We focused our experiments on *Mycobacterium smegmatis*, an experimentally tractable surrogate for *M*. *tuberculosis* with respect to rifampicin—a frontline drug in TB treatment [23]. The microdialyser has a micro-sized cell culture chamber that is 200 picoliters (pL) in volume to approximate the ~5pL volume of the membrane-bound compartment of human macrophages [24]. Thus upon inoculation of a microdialyser culture chamber with ~5 mycobacteria, the cell density immediately exceeds 10^7^ cells/ml and verges toward the effective cell density of an intra-macrophage mycobacterium, ~10^8^ cells/ml [24].

## Materials and Methods

### Microfluidic device design and fabrication

The microdialyser chip was fabricated out of the silicone elastomer polydimethylsiloxane (PDMS) (General Electric RTV 615) using multi-layer soft lithography, as described previously [25]. Up to 120 microdialyser units can run in parallel on each chip.

### The microdialyser reader

Mycobacteria were cultivated in growth chambers within the microdialyser chip (S1 Fig) which was positioned for live-cell imaging on an Olympus IX81 inverted microscope furnished with a PRIOR Scientific XYZ motorized stage system (Wirsam Scientific Precision Equipment (Pty) Ltd., Pinetown, South Africa). The motorized stage system enabled documentation of multiple simultaneous microdialyser cultures on a single chip. Imaging was done using a Plan Fluor 40X 0.6NA objective. Digital images were captured by a Hamamatsu digital CCD ORCA-R2 camera (Wirsam Scientific Precision Equipment (Pty) Ltd., Pinetown, South Africa). LabVIEW software was used to control the synchronized operation of these components and chip valve actuation.

### Media, strains and growth conditions

*M*. *smegmatis* strain mc^2^155 was received from Bill Jacobs (Albert Einstein College of Medicine). *M*. *smegmatis* Δ*ftsEX*—gift from Eric Rubin (Harvard University) comprised of an *M*. *smegmatis* mc^2^155 strain with the *ftsEX* gene deleted and were hypersensitive to the RNA polymerase inhibitor, rifampicin, with a minimum inhibitory concentration of 1μg/ml [26]. *E*. *coli* Top10F’ cells were purchased from Invitrogen. Conventional cell cultures were performed with 10 ml of 7H9 broth in tissue culture flasks at 37°C with shaking at 100 rpm and growth was determined by measuring the turbidity of the cultures at 600 nm (OD_600_) twice daily unless otherwise stated. *M*. *smegmatis* cells in the microdialyser were grown in 7H9 broth at 37°C. The *M*. *smegmatis mmpL11* mutant was described previously [27].

*M*. *smegmatis* precultures were prepared by inoculating a 10ml medium (7H9 broth) sample with cells from a frozen stock and culturing at 37°C with shaking at 100 rpm until an OD_600_ reading of 0.8 was reached. The cell culture was centrifuged at 2500 rpm for one minute to cause the large cell clusters to gather towards the bottom of the tube. To load the cells into the microdialyser chip, first ~500μL of the supernatant cell suspension was aspirated into a piece of tygon tubing (0.02 OD X 0.06 ID, Cole Parmer) by suction using a 1 ml syringe. Next, the free end of the tygon tubing was connected to a cell input port on the microdialyser chip (see S1 Fig), using a stainless steel pin as an adaptor between the chip and the tygon tubing. The cells were introduced into each growth chamber by flowing the cell suspension from the tygon tubing through each chamber: in via an open inlet and out via an open outlet. Accordingly, cells were trapped in each growth chamber once the inlet and outlet of the chamber was subsequently closed, by actuating the appropriate valves on the chip. On average, cells were trapped in each chamber at a uniform cell density of ~2×10^7^ cells/ml. Thus, on average, the starting number of cells per growth chamber in the 200, 500, 1200 and 1700pL growth chambers was 5, 12, 30 and 42 cells respectively. Upon microdialyser inoculation, cells were incubated in the microdialyser growth chambers by maintaining the chip at 37°C using a Solent Scientific microscope incubation chamber heating system (Wirsam Scientific Precision Equipment (Pty) Ltd., Pinetown, South Africa).

### The microdialyser operation process

**Fig 1:**
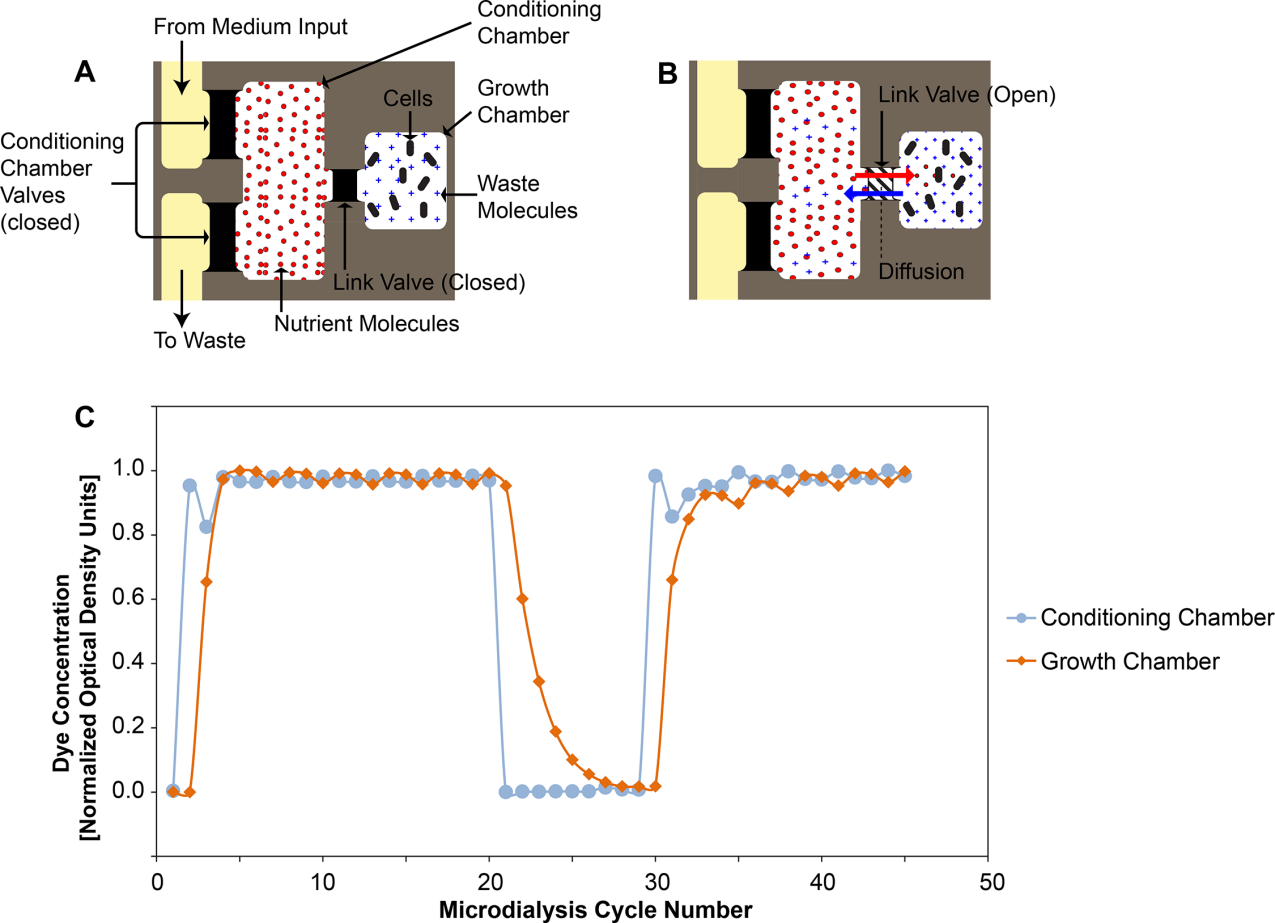
The microdialyser system. **(A)** Schematic diagram of a microdialyser unit with elements such as the growth and conditioning chambers, separated by a link valve labelled. **(B)** Once the link valve is open, passive diffusive exchange occurs between the growth and conditioning chambers: nutrient molecules (represented by red dots) diffuse into the growth chamber while mycobacterial metabolic waste products (blue crosses) diffuse into the conditioning chamber. **(C)** Functional illustration of diffusive exchange in the microdialyser showing the dye concentration in the growth chamber (orange diamonds) and the conditioning chamber (light blue circles). The fluid in the conditioning chamber is cycled between DYE (cycles 2–20 and 30–45) and WATER (cycles 1; 21–29). The dye concentration in the growth chamber depends on the concentration in the conditioning chamber. This result was reproduced over 160 times in 9 different chips.

The microdialyser uses a microdialysis scheme to periodically introduce fresh nutrients and remove waste from a captive population of mycobacteria through diffusive exchange, mimicking the passive bidirectional exchange of pro- and anti-mycobacterial factors across the macrophage membrane (Fig 1). The 200pL mycobacterial growth chamber of the microdialyser is connected via one or more link valves to an adjacent conditioning chamber that stores fresh medium (Fig 1A). Periodically (typically, hourly), the link-valves are opened for 60 seconds to allow small molecules to freely diffuse between the two chambers down their respective concentration gradients: fresh nutrients diffuse into the growth chamber while mycobacterial metabolic waste products diffuse into the conditioning chamber (Fig 1B). The relatively large size and non-motility deter the mycobacterial cells from exiting the growth chamber when the link valves are opened. Next, the conditioning chamber is refilled with fresh medium and awaits the next microdialysis step. We calibrated the diffusive exchange functionality of the microdialyser using colorimetric assays and demonstrated that on average, the microdialyser replaced the growth chamber fluid within six microdialysis steps (Fig 1C).

### Colorimetric assays

The food dye used in the colorimetric microdialyser characterization assay was obtained from McCormick & Co., Hunt Valley, MD. The dye concentration in the microdialyser was determined based on the average pixel value of optical micrographs of a region within the growth chamber or the conditioning chamber during a series of microdialysis steps. The camera pixel value (**P**) is linear with respect to transmission (**T)**, which is the anti-log of the negative of the optical density (**OD**) [28], as depicted in the equations below.

$$\mathrm{OD}=-{log}_{10}\left(T\right)$$

$$\mathrm{OD}≅-{log}_{10}\left(P\right)$$

### Microscopic cell surface density

The microdialyser architecture is such that all the cells dwell in a chamber ≤10 μm high, equivalent to the focal depth of the Plan Fluor 40X 0.6NA objective. We assessed bacterial growth in the microdialyser by enumerating the fraction of pixels occupied by *M*. *smegmatis* cells in images of microdialyser culture chambers (or cell surface density—a dimensionless quantity with a maximum value of 1), at different time points. We developed image-processing algorithms written in Matlab to determine the cell surface density in each picture. The motorized stage system enabled documentation of multiple simultaneous microdialyser experiments on a single chip.

## Results and Discussion

**Fig 2:**
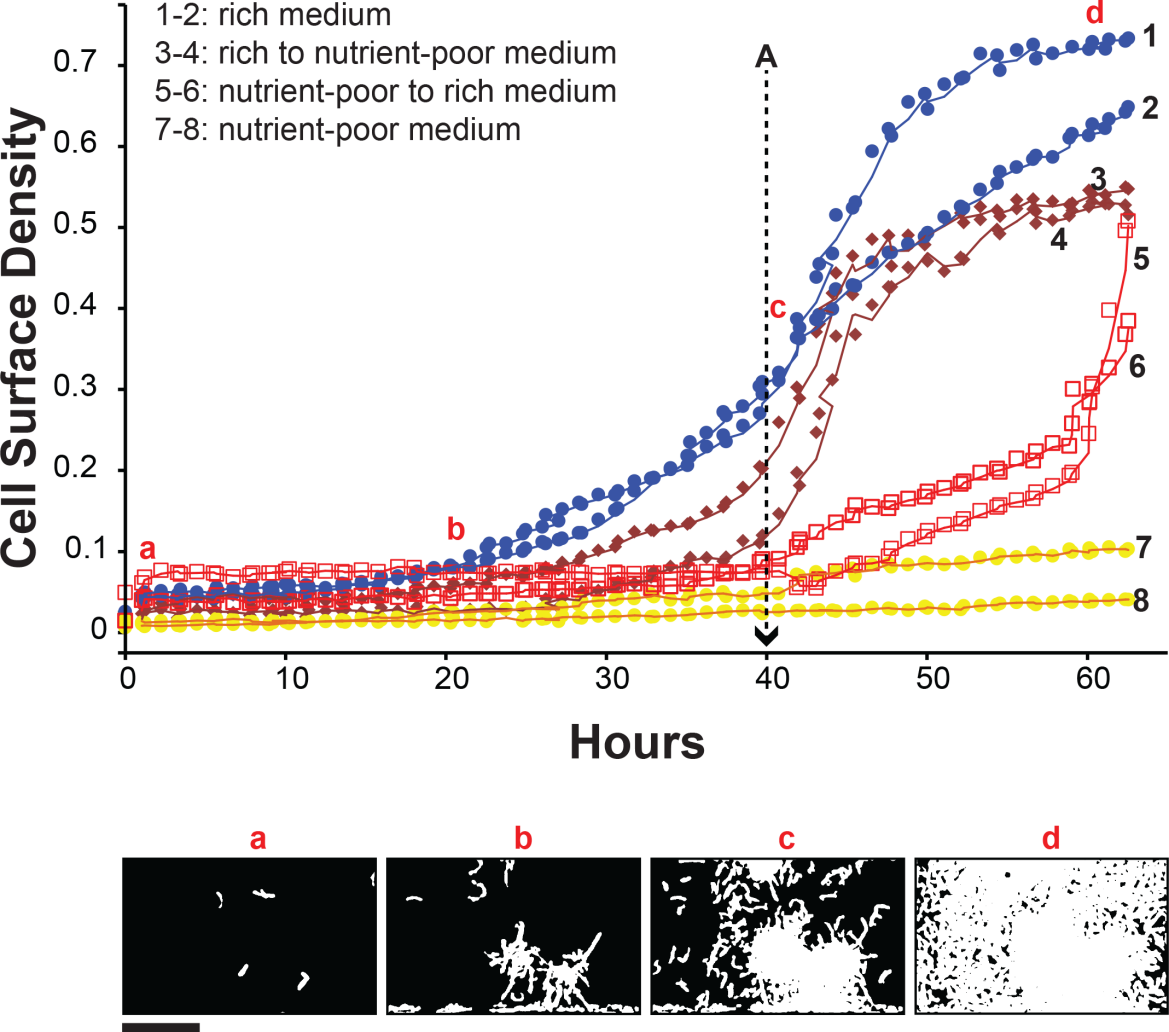
Functional validation of the microdialyser system. Typical growth curves of *M*. *smegmatis* mc^2^155 cells in a microdialyser (growth chamber volume, 200pL) with the medium switching from nutrient-poor to nutrient-rich and vice versa. Initially, cultures 1 to 4 are grown in rich medium (7H9), and 5 to 8 in nutrient-poor medium (spent 7H9 medium harvested from *M*. *smegmatis* cell cultures that have reached stationary phase). At 40 hours (point A), the rich medium in cultures 3 and 4 was replaced with nutrient-poor medium using microdialysis, which slowed down growth in these cultures relative cultures 1 and 2 that remained rich. Conversely, at point A, the nutrient-poor medium in cultures 5 and 6 was replaced with rich medium, resulting in faster growth in these cultures relative cultures 7 and 8 that remained nutrient-poor. Cultures 1 to 8 were cultivated simultaneously on the same chip at 37°C. Each condition was demonstrated in at least three cultures. Bottom panels (a to d) show typical area maps of the cells in the growth chamber of microdialyser 1 at the corresponding points from which the cell surface density is determined. Scale bar, 50μm.

At full operational capacity, the microdialyser chip can support 120 microdialyser culture units that operate independently, with each culture monitored in situ by optical microscopy to provide automated, real-time, non-invasive measurement of cell density. Mycobacterial growth was assessed from microscopic images of each growth chamber by enumerating the fraction of pixels occupied by bacterial cells (or cell surface density—a dimensionless quantity with a maximum possible value of 1) at different time points. Microdialyser growth curves followed the trend of typical mycobacterial growth: upon inoculation, a typical culture began with a lag period, followed by an exponential growth phase that gave way to a stationary phase. To functionally validate the effectiveness of the microdialyser in modulating the growth environment of its captive mycobacterial population, we demonstrated the ability to speed up or slow down the mycobacterial growth by switching the growth chamber medium from nutrient-poor to nutrient-rich and vice versa (Fig 2).

**Fig 3:**
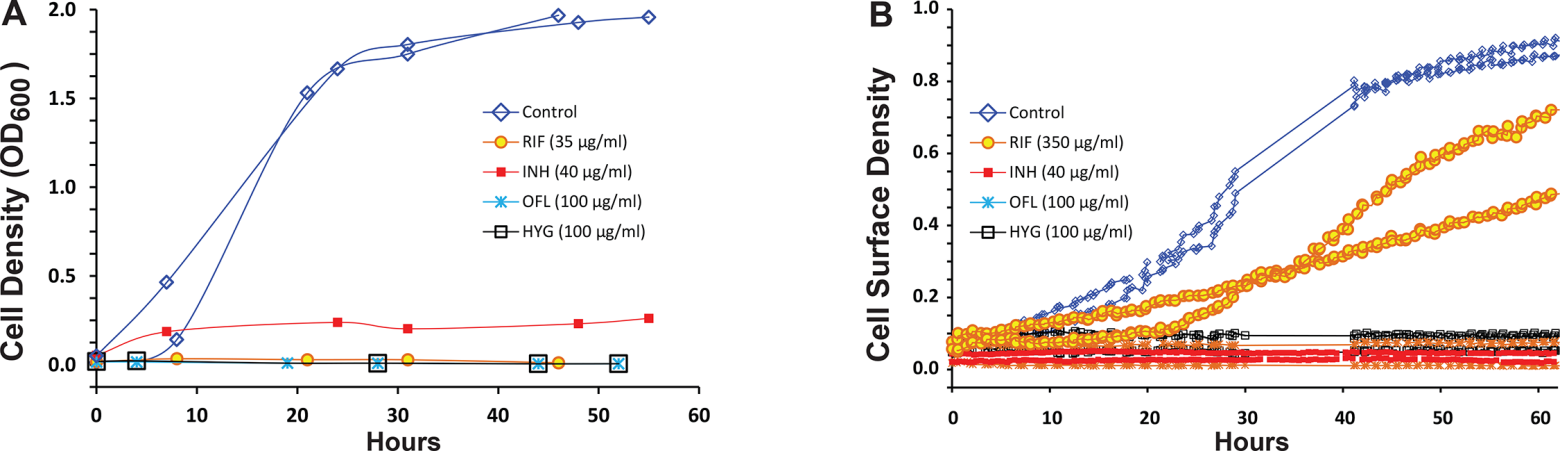
Drug susceptibility of *M*. *smegmatis* mc^2^155 cells ON- and OFF-chip. **(A)** mc^2^155 tissue culture flask growth curves illustrating the growth-inhibitory effect of rifampicin (35μg/ml), isoniazid (40μg/ml), ofloxacin (100 μg/ml) and hygromycin (100μg/ml). All measurements were performed in duplicate. **(B)** Microdialyser drug susceptibility of mc^2^155 cells growing with rifampicin (350μg/ml), isoniazid (40μg/ml), ofloxacin (100 μg/ml) and hygromycin (100μg/ml) in 200 picoliter growth chambers. All the drugs except rifampicin had a strong growth-inhibitory effect at the concentrations indicated in the microdialyser. Over 10 control cultures performed exhibited positive growth. Hygromycin and ofloxacin inhibited growth in 5 distinct cultures per drug. Isoniazid inhibited growth in 8 of 11 cultures. Two of the isoniazid cultures had minimal growth and in one growth was more significant (S3 Fig). Rifampicin cultures had significant growth in 11 of 12 cultures (S4 Fig).

Conventional liquid phase cultures of *M*. *smegmatis* mc^2^155 cells were treated with a series of antimycobacterial drugs, including rifampicin (35μg/ml), isoniazid (40μg/ml), ofloxacin (100 μg/ml) and hygromycin (100μg/ml). Growth was inhibited in conventional cultures by these drugs at the indicated concentrations (Fig 3A). Growth in microdialyser cultures was inhibited by the drugs with the exception of rifampicin, which had minimal growth inhibitory effect even at 350 μg/ml—which is 10× the concentration that inhibited growth in the conventional liquid phase cultures, ~40× the minimum inhibitory concentration (MIC) and ~10× the minimum bactericidal concentration (MBC) of rifampicin for mc^2^155 cells [29, 30] (Fig 3B). For comparison, rifampicin at 350 μg/ml inhibited growth of *Escherichia coli* cells in microdialyser cultures and maintained its antibiotic potency for more than 200 hours, demonstrating that there was sufficient penetrance of the rifampicin into the microdialyser reactors (S2 Fig). This result also indicated that the rifampicin tolerance phenotype was specific to *M*. *smegmatis* and absent in *E*. *coli*. Given the small number of *M*. *smegmatis* cells present in the growth chamber (not exceeding 20) at the time the rifampicin resistance first appeared, a simple mutation rate versus population size argument excludes the possibility of a mutational cause of resistance to rifampicin. Because rifampicin is a front line drug in TB treatment, we sought to better characterize and further elucidate mechanisms underlying the rifampicin tolerance phenotype.

**Fig 4:**
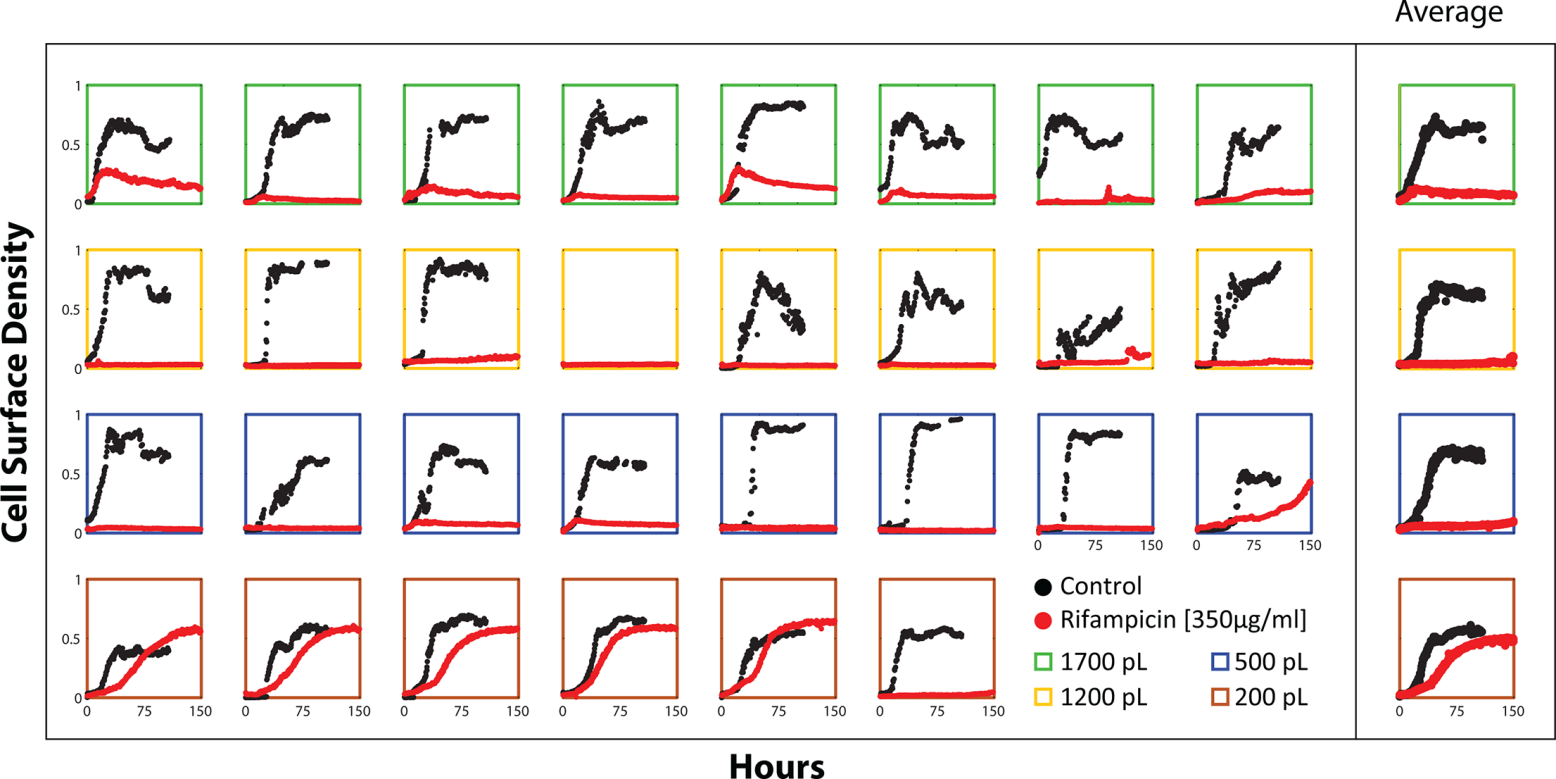
Growth dynamics of *M*. *smegmatis ΔftsEX* cells with or without rifampicin (350 μg/ml) across the various microdialyser growth chambers sizes: 1700pL (green boxes), 1200pL (yellow boxes), 500pL (blue boxes) and 200pL (brown boxes). The growth curves in the right-most column represent the average cell density of the individual reactors shown on the left. In drug-free medium, the cells exhibited positive growth across all the growth chamber volumes as indicated (black growth curves). With rifampicin, growth in five of six of the 200pL reactors was only slightly affected with the exception of one of the growth chambers in which the cells did not grow. Growth in the larger reactors (eight 1700pL, eight 1200pL and seven 500pL) was inhibited (red curves), except in one of the 500pL growth chambers. The cultures under each drug condition were cultivated simultaneously on the same chip at 37°C.

To investigate the role of confinement in the rifampicin resistance of microdialyser cell populations, we fabricated a new chip with growth chambers of various sizes: 200pL, 500pL, 1200pL and 1700pL (S1 Fig). Colorimetric assays ascertained that the diffusive penetrance of the microdialyser process was similar across all growth chamber sizes (S5 Fig). In addition, we obtained an Δ*ftsEX* mutant of *M*. *smegmatis* that is particularly hypersensitive to rifampicin with a MIC of 1μg/ml [26]. *M*. *smegmatis* Δ*ftsEX* cells grew similarly well in the various growth chamber sizes in drug-free medium. However, although *M*. *smegmatis* Δ*ftsEX* demonstrated resistance to rifampicin at 350 μg/ml in the 200pL cultures, the drug inhibited growth in the larger (500pL, 1200pL and 1700pL) cultures (Fig 4). Thus, the rifampicin resistance phenotype was dependent on the size of the growth chamber: appearing when the mycobacteria were cultured in the smallest (200pL) growth chambers and disappearing in the bigger reactor volumes. Wild type mc^2^155 *M*. *smegmatis* cells growing in various sized growth chambers had a similar pattern of drug tolerance behavior when exposed to 350μg/ml of rifampicin (S4 Fig).

**Fig 5:**
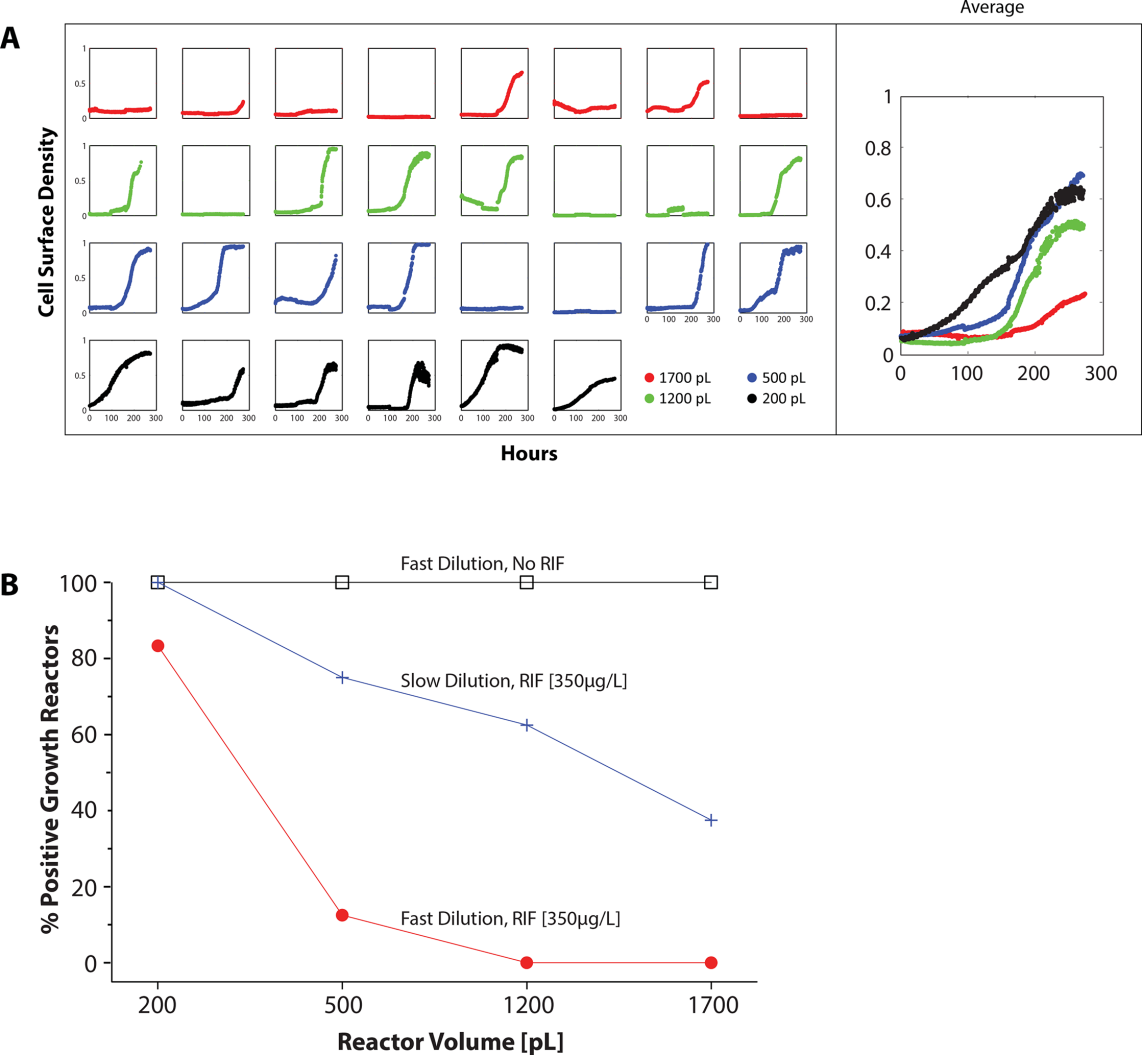
(A) Growth of *M*. *smegmatis ΔFtsEX* cells with rifampicin [350 μg/ml] with the time interval between consecutive microdialysis steps doubled to 2 hours—across the various microdialyser growth chambers sizes—1700pL (red curves), 1200pL (green), 500pL (blue) and 200pL (black). The growth curves on the right represent the average cell density of the individual reactors shown on the left. With the slower dilution rate, the cell populations in the larger reactors become more tolerant to rifampicin in accordance to the reactor volume: the fraction of reactors with growth decreases as the volume increases. All cultures were cultivated simultaneously on the same chip at 37°C. (B) The percentage of microdialyser cultures with positive growth at different dilution rates with rifampicin (350μg/ml) or in drug-free medium. At a dilution (microdialysis) interval of one hour, all drug-free cultures (black boxes) reactors registered positive growth independent of culture volume. Five of six 200pL cultures and among the larger cultures, only one of the eight 500pL cultures tolerated rifampicin (red circles). With the dilution interval doubled to two hours, the yield of the drug-containing cultures (blue crosses) increased albeit in a volume-dependent fashion, whereby the fraction of cultures with positive growth decreased as the culture volume increased. The cultures were cultivated at 37°C.

One potential cause of volume-dependent rifampicin tolerance may be due to increased bacterial density in space-confined environments through quorum sensing. In the generic model of quorum sensing [31, 32], bacterial cells secrete signaling molecules called autoinducers whose concentration in the surrounding medium increases with cell density. At low cell density, the autoinducer molecules produced at a basal level diffuse out of the cell, and are ultimately lost to the environment. As the cell density increases, the autoinducer concentration reaches a threshold concentration and triggers a transcriptional response that results in increased expression of virulence determinants [33, 34], upregulation of biofilm formation [35–37] or entry into stationary phase [38, 39]—that is unattainable with low cell density. Large volume cultures require a proportionately large cell population to elicit quorum sensing behavior. However, in a sufficiently small growth environment, a few cells can elicit quorum sensing behavior more efficiently because the autoinducer molecules produced are kept in close proximity by the boundaries of the confined growth compartment and can therefore accumulate faster [40–42]. In a pattern consistent with quorum sensing control, when the time interval between consecutive microdialysis steps was doubled to two hours, the larger microdialyser cultures became more tolerant to rifampicin in a volume-dependent fashion, whereby the fraction of cultures with positive growth increased as the culture volume decreased (Fig 5). Notably, an inevitable consequence of the slower dilution rate was a reduced cellular growth rate due to the proportionately less frequent infusion of fresh nutrients. Bacteria growing with slower growth rates may be more tolerant to rifampicin, and therefore these cells experiencing a slower dilution rate may have a higher frequency of recovery. We measured the bacterial growth rates in the various growth chamber sizes in drug-free medium and found them to be similar (S6 Fig). Therefore, whereas slower growth rate may be a probable cause of the higher recovery frequency at the slower dilution rate, it does not explain the differential drug tolerance in the various growth chamber sizes. This pattern of expression is consistent with quorum sensing and may allow us to further study this system in mycobacteria.

**Fig 6:**
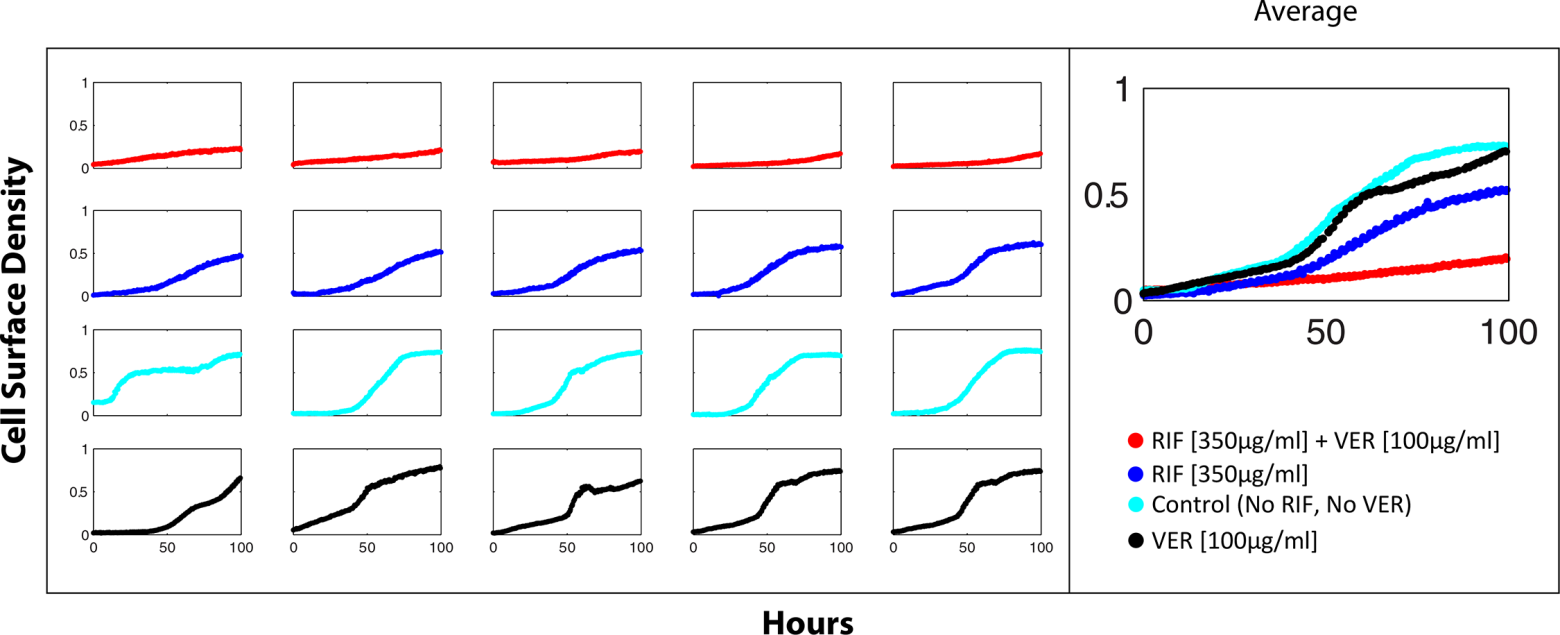
Efflux inhibition reduces drug tolerance. Effect of verapamil (100μg/ml) on 200pL ΔFtsEX cultures with or without rifampicin (350 μg/ml). The right-most growth curves represent the average cell density of the individual reactors shown in each row on the left. The control (cyan growth curves), rifampicin only (blue) and verapamil only (black) cultures registered positive growth. Rifampicin tolerance was dramatically reduced in cultures that also contained verapamil (red). All cultures were cultivated simultaneously on the same chip at 37°C.

Studies show that microbial efflux pumps can confer epigenetic resistance to antibiotics by enabling bacterial cells to extrude the drug molecules intended to kill them [18, 43–46]. We explored efflux activity as a potential underlying mechanism for rifampicin tolerance in the 200pL using the efflux inhibitor approach, which is widely used to indicate efflux activity [18, 47–49]. *M*. *smegmatis* Δ*ftsEX* cells were cultured with rifampicin in the presence of verapamil—a mycobacterial efflux inhibitor [50]. As per the efflux inhibitor approach, dramatically reduced growth of the cells in the presence of verapamil suggested that confinement-induced drug tolerance was mediated by efflux mechanisms (Fig 6).

**Fig 7:**
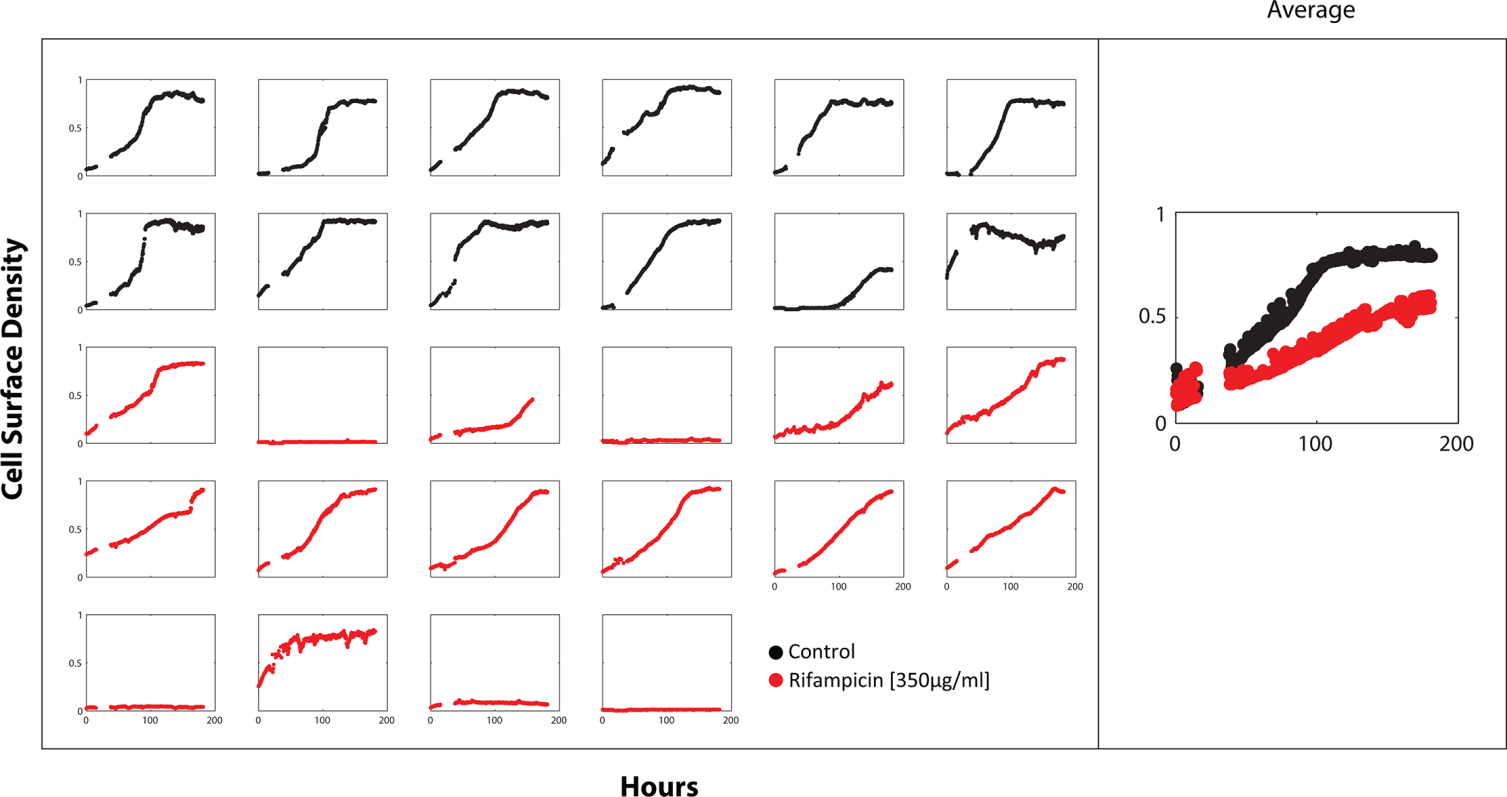
Loss of MmpL11 function reduced resistance to rifampicin in the 200pL cultures. Effect of rifampicin (350 μg/ml) on the growth of *M*. *smegmatis mmpL11* mutants in 200pL cultures. The growth curves in the right-most column represent the average cell density of the individual reactors shown on the left. All 12 (100%) mutant drug-free cultures grew (black growth curves). However, only 11 of 16 (69%) mutant cultures grew in rifampicin medium and growth in these cultures was generally slower. All cultures were cultivated simultaneously on the same chip at 37°C.

The discrepancy in drug sensitivity between the small and large volume microdialyser cultures can be exploited to investigate the genetic markers underlying confinement-induced drug tolerance. As a proof of principle, we investigated MmpL11, a cell wall lipid transport protein that contributes to biofilm formation in *M*. *smegmatis* [51]. In drug-free 200pL cultures, an *M*. *smegmatis* mutant lacking *mmpL11* functionality grew in all 12 reactors (Fig 7). However, only 11 of 16 mutant cultures registered growth in rifampicin medium, notwithstanding that the growth rate was slower in these cultures. Loss of MmpL11 function reduced resistance to rifampicin in the 200pL cultures, suggesting that MmpL11 protein contributes to confinement-induced rifampicin tolerance. It should be noted that there is no difference in rifampicin MIC between the wild-type *M*. *smegmatis* and *mmpL11* mutant during typical axenic culture. While bacteria cultured in the microdialysers are not forming biofilms per se, these results suggest that similar changes in the mycobacterial cell wall lipid composition occur in the confined space of a micro reactor as in a biofilm that result in a drug-tolerant phenotype. In the absence of MmpL11, *M*. *smegmatis* is unable to establish this drug-resistant state.

**Fig 8:**
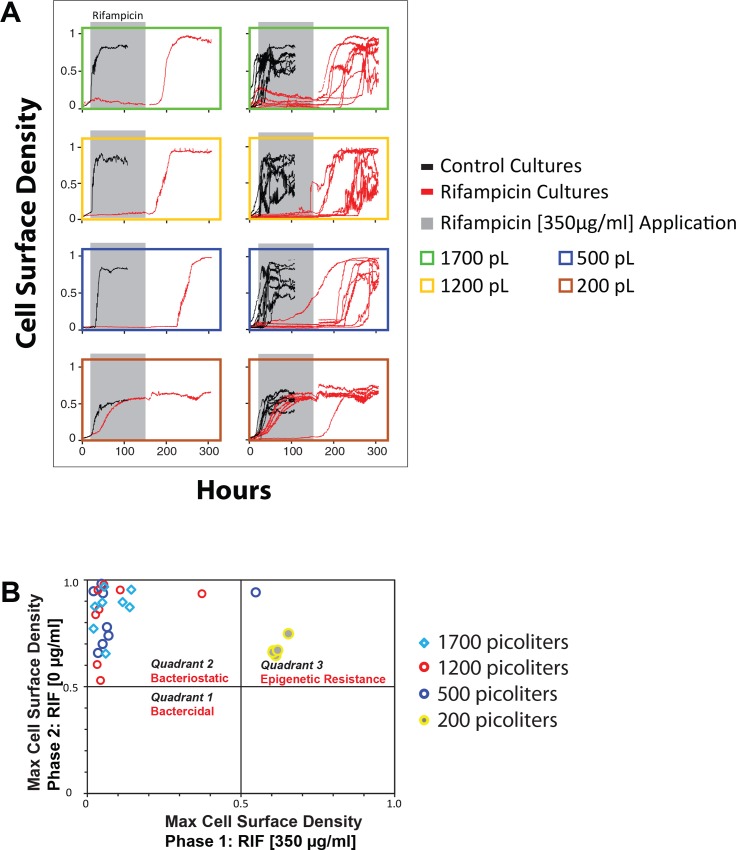
(A) Use of the microdialyser system to resolve the bacteriostatic/bactericidal credentials of antimicrobial drugs. The graphs in the left column represent a typical growth curves for each growth chamber size and those in the right column represent a set of growth curves for the same growth chamber volume. The cultures represented by the red curves were first cultured in drug-free medium for 15 hours. Using the microdialysis process, at 15 hours, rifampicin (350 μg/ml) was introduced to these cultures and then withdrawn at 150 hours. Although microbial growth in the bigger volume reactors—seven 500, eight 1200 and eight 1700pL—was effectively suppressed when rifampicin was applied, cell growth resumed when the drug was withdrawn, suggesting that rifampicin was bacteriostatic under these conditions. The cells in one of the eight 500pL maintained slow growth in the presence of the drug. Microbial growth in five of six small (200pL) growth chambers was only slightly decreased by the drug and suppressed in one of the reactors in which the drug was bacteriostatic. Control cultures are depicted by black curves demonstrating consistently positive growth in drug-free medium across all growth chamber sizes. (B) A system for distinguishing the bacteriostatic/bactericidal credentials of antimicrobial drugs. The X-axis represents the cell density reached by ΔFtsEX cells in the reactors shown above (Fig 8A) after culturing for 150 hours in medium with rifampicin (350 μg/ml) during the first phase of culture. The Y-axis represents the new cell density attained in the same reactors after 150 additional hours of culturing in medium without rifampicin. Accordingly, data points in quadrants 1, 2 and 3 represent reactors in which the cells are dead, static or resistant respectively. In general *M*. *smegmatis* Δ*ftsEX* cells were resistant to rifampicin in the 200pL reactors and static in the rest.

Unlike bactericidal antibiotics, bacteriostatic drugs ultimately depend upon the immune system for sterilization, and are therefore poor treatment options where the immune system is compromised [52]. Distinguishing between bacteriostatic and bactericidal action of antimicrobial drugs can be cumbersome using conventional drug susceptibility testing but the microdialyser can rapidly resolve this distinction for antimicrobial agents. Using the microdialyser process, we substituted the rifampicin-containing medium with drug-free medium without otherwise perturbing the cells in the non-growing cultures. Upon withdrawal of rifampicin via microdialysis, growth resumed in all the larger reactors (Fig 8), suggesting that the growth-inhibitory effect of rifampicin was of the bacteriostatic kind. It is worth noting that some conventional studies have indicated that rifampicin is bactericidal to *M*. *smegmatis* cells at 32 μg/ml [30].

## Conclusions

A physical cell culture system that mimics confinement of mycobacteria such as in a macrophage during infection enabled us to investigate confinement-induced drug tolerance in *M*. *smegmatis* cells. We uncovered an epigenetic rifampicin-resistance phenotype that was dependent on the size of the growth chamber: appearing when the mycobacteria were cultured in the small (200pL) growth chambers and disappearing in the bigger reactor volumes (500pL, 1200pL and 1700pL). The drug-tolerant phenotype was mediated in large part by efflux mechanisms, which were induced in confined growth environment. The confinement-induced drug tolerance observed in the microdialyser has similarities with the macrophage-induced tolerance that others have reported in intramacrophage mycobacterial species [18]. To the degree that confinement is a significant common factor for mycobacteria replicating in small microdialyser reactors or macrophages, macrophage-induced drug tolerance may be an effect of confined growth in addition to other macrophage specific mechanisms.

In vitro drug susceptibility tests performed using conventional cell culture systems, which are the basis for drug prescriptions, are notoriously poor predictors of treatment outcomes in human TB [53, 54]. Our results suggest that the volume difference between the in vitro and in vivo (intramacrophage) growth compartments for *M*. *tuberculosis*, together with its implications for epigenetic drug resistance, may be a contributor to the apparent incongruity between drug susceptibility tests and treatment outcomes. Indeed, unlike the mycobacteria freshly inoculated into a conventional culture vessel, intramacrophage mycobacteria, which comprise a significant portion of TB infection [16], experience a space-confined growth environment that our experiments suggest can induce drug tolerance. Confinement-induced drug tolerance in *M*. *tuberculosis* may contribute to persistence in tuberculosis patients, where drugs that are rapidly potent in vitro require prolonged administration to achieve comparable effects. The microdialyser may thus provide an appropriate paradigm for research on therapeutic interventions aimed at rapidly neutralizing the drug-tolerant mycobacteria that currently prolong treatment in human TB.

## Supporting Information

S1 Fig Design and operation of the microdialyser with multiple volume reactors. **(A)** Optical micrograph of the microdialyser chip showing a single module. Scale bar, 0.5mm. The module features 8 medium input ports, 5 cell input ports, 10 output ports and 30 individually addressable microdialyser culture units: six 200-, eight 500-, eight 1200 and eight 1700-picoliter cell culture chambers. The blue rectangular box indicates the region depicted in B, C and D. **(B)** Optical micrograph showing three microdialysers in a row to illustrate the main aspects of the microdialysis scheme. Elements such as the growth chamber, conditioning chamber and link valves are labelled (see Fig 1). With the link valve closed, the conditioning chamber of the middle microdialyser unit is filled with blue dye (representing fresh medium). Scale bar, 0.3mm. **(C)** Once the link valve is open, diffusive exchange between the growth and conditioning chambers occurs. **(D)** After a series of (typically six) microdialysis steps, the growth chamber fluid is completely replaced with the fluid introduced via the conditioning chamber.
(PNG)

S2 Fig *E*. *coli* cells are susceptible to rifampicin in 200pL microdialyser reactors. Growth of *E*. *coli* Top10 F’ cells in 200pL microdialyser reactors with switching between medium with and without rifampicin. The red curves represent microdialyser *E*. *coli* cultures that were initially drug-free (0 to 110hrs). Addition of rifampicin (350 μg/ml) at 110hrs (point A) resulted in a dramatic decrease in the cell population. The blue curves represent *E*. *coli* cultures that initially contained 350 μg/ml of rifampicin (0 to 110hrs). Removal of the drug at point A led to growth recovery in 2 of 12 cultures. All cultures were cultivated on the same chip in LB medium at 37°C.
(EPS)

S3 Fig Isoniazid susceptibility of *M*. *smegmatis* mc^2^155 cells in microdialysor reactors. Microdialyser drug susceptibility of mc^2^155 cells growing with isoniazid (40μg/ml) in 200pL growth chambers. Isoniazid inhibited growth in 8 of 11 cultures incubated in two chips. Two of the isoniazid cultures had minimal growth (reaching a cell surface density of 0.2) and in one growth was more significant (reaching a cell surface density of 0.4).
(EPS)

S4 Fig Growth dynamics of *M*. *smegmatis* mc^2^155 cells with or without rifampicin (350 μg/ml) across the various microdialyser growth chambers sizes. In drug-free medium (black curves), the cells exhibited positive growth across all the growth chamber volumes as indicated—1700pL, growth in 8 of 8 cultures; 1200pL, growth in 8 of 8 cultures; 500pL, growth in 7 of 7 cultures; and 200pL, growth in 6 of 6 cultures. With rifampicin (red curves), growth in the larger reactors was inhibited—1700pL, growth in 0 of 8 cultures; 1200pL, growth in 0 of 8 cultures; and 500pL, growth in 0 of 8 cultures. In the 200pL cultures with rifampicin, there was significant growth in 11 of 12 growth chambers.
(EPS)

S5 Fig Functional illustration of diffusive exchange in the multi-volume microdialyser showing the dye concentration in the various growth chamber volumes and the conditioning chamber. The formulation in the conditioning chambers is cycled between DYE (cycles 2–7 and 13–19) and WATER (cycles 1; 8–12). The dye concentration in the various growth chambers is determined by the formulation in the conditioning chamber. The chamber-to-chamber variations in dye concentration are attributable to minor height differences in the growth chambers.
(EPS)

S6 Fig Exponential growth rates for *M*. *smegmatis ΔFtsEX* cells growing in various growth chamber sizes in drug-free medium. Various growth rates were measured for bacteria growing in the drug-free microdialyser cultures depicted in Fig 4, however there was no significant difference in growth rates across the various growth chamber sizes.
(EPS)
