# Supplementary figures and images for: Confinement-Induced Drug-Tolerance in Mycobacteria Mediated by an Efflux Mechanism
Source: PLoS One. 2015 Aug 21;10(8):e0136231. doi: 10.1371/journal.pone.0136231 (PMC4546595; doi:10.1371/journal.pone.0136231)

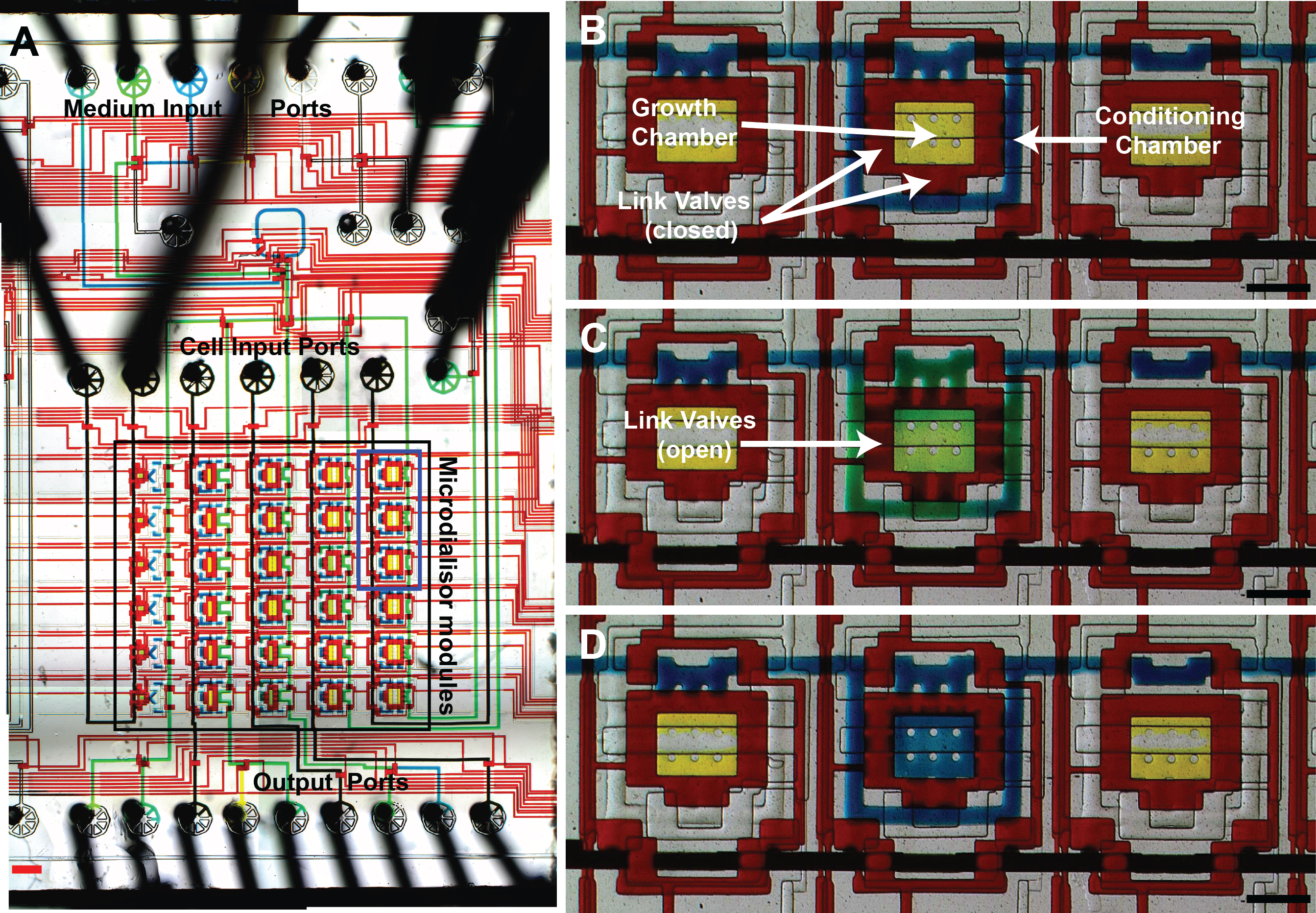

Supplement: S1 Fig — (A) Optical micrograph of the microdialyser chip showing a single module. Scale bar, 0.5mm. The module features 8 medium input ports, 5 cell input ports, 10 output ports and 30 individually addressable microdialyser culture units: six 200-, eight 500-, eight 1200 and eight 1700-picoliter cell culture chambers. The blue rectangular box indicates the region depicted in B, C and D. (B) Optical micrograph showing three microdialysers in a row to illustrate the main aspects of the microdialysis scheme. Elements such as the growth chamber, conditioning chamber and link valves are labelled (see Fig 1). With the link valve closed, the conditioning chamber of the middle microdialyser unit is filled with blue dye (representing fresh medium). Scale bar, 0.3mm. (C) Once the link valve is open, diffusive exchange between the growth and conditioning chambers occurs. (D) After a series of (typically six) microdialysis steps, the growth chamber fluid is completely replaced with the fluid introduced via the conditioning chamber. (PNG) [file pone.0136231.s001.png]
